# Application Value of Minimally Invasive Percutaneous Dilational Tracheostomy for ICU Critical Patients

**DOI:** 10.12669/pjms.36.2.594

**Published:** 2020

**Authors:** Hongmei Gan, Aihong Dong, Haiyan Xu

**Affiliations:** 1 Hongmei Gan ICU, Binzhou People’s Hospital, Shandong, 256610, China; 2 Aihong Dong Department of General Practice, Binzhou People’s Hospital, Shandong, 256610, China; 3 Haiyan Xu Department of Endoscope, Binzhou People’s Hospital, Shandong, 256610, China

**Keywords:** Percutaneous dilational tracheostomy, Conventional tracheostomy, Intensive care unit

## Abstract

**Objective:**

To find out the application value of minimally invasive percutaneous dilational tracheostomy in critically ill patients in intensive care unit (ICU).

**Methods:**

One hundred and forty critically ill patients who underwent tracheostomy in ICU of our hospital were included in the study from August 2016 to December 2017. They were divided into an observation group and a control group by random number table method, 70 in each group. The control group received conventional tracheotomy, while the observation group received percutaneous dilational tracheostomy. The operation time, incision length, amount of intraoperative bleeding and healing time of incision were compared between the two groups, and the changes of vital signs and complications after operation were recorded. The family members of the patients signed the informed consent.

**Results:**

The operation time, healing time and incision length of the observation group were (9.92±4.13) min, (1.31±0.21) cm and (6.91±0.72) d respectively, shorter than (24.09±6.82) min, (3.40±0.65) cm and (67.48±0.61) d in the control group, and the differences were statistically significant (P<0.05). The amount of intraoperative bleeding of the observation group was (7.81±1.83) mL, less than (16.34±2.83) mL in the control group; the difference was statistically significant (P<0.05). The heart rate and oxygen saturation of the observation group before and during the operation were not significantly different (P>0.05). The heart rate of the control group during the operation was significantly higher than that before the operation (P<0.05); the oxygen saturation of the control group before and during the operation had no significant difference (P>0.05). The incidence of complications in the observation group was 24.3%, which was significantly lower than that in the control group (55.7%, X^2^=8.279, P=0.014).

**Conclusion:**

Minimally invasive percutaneous dilational tracheostomy has advantages of small trauma, less infection and beautiful incision, and it will not increase postoperative complications. It is of great value in the treatment of ICU critical patients.

## INTRODUCTION

Critically ill patients often have dangerous condition and poor prognosis. Cardiac arrest may occur after 4-6 minutes of asphyxia because of dyspnea caused by laryngeal obstruction or lower respiratory tract secretion retention, and irreversible damage can occur after 4-6 minutes of cerebral ischemia and hypoxia; therefore, early diagnosis and treatment should be given to critically ill patients.^1^,^2^ The key of rescuing critically ill patients is to rapidly establish effective airway and keep smooth breath, which is especially important for critically ill patients with infection in intensive care unit (ICU).^3^ In the past, tracheotomy was often used to establish artificial airway for patients. Although it can remove airway obstruction, improve the situation of respiratory obstruction and improve the prognosis of patients, the large trauma and multiple postoperative complications limited its clinical application.^4^-^7^ The incidence of complications and mortality rate among patients undergoing tracheostomy is reported as 6%~66% and 0%~5.3% abroad.^8^ Percutaneous dilational tracheostomy is a minimally invasive surgical technique developed in recent years.^9^,^10^ It was first reported by Ciaglia et al. in 1985.^11^ It has been gradually applied in China because of its advantages of simple operation and small trauma, but there is no large-sample study.^8^,^9^ In order to compare the effect of percutaneous dilatational tracheostomy and conventional tracheostomy and to analyze the feasibility of percutaneous dilatational tracheostomy in intensive care unit (ICU), 70 critically ill patients were treated with percutaneous dilational tracheostomy in this study.

## METHODS

According to the random principle, 140 critically ill patients who underwent tracheostomy in ICU of Binzhou People’s Hospital from August 2016 to December 2017 were selected as the research subjects. They were divided into observation group and control group by the random number table method, 70 each group. The observation group consisted of 39 males and 31 females, aged 20-65 years, with an average age of (48.6±5.7) years. There were 22 cases of cardiopulmonary resuscitation, 20 cases of craniocerebral trauma and cerebrovascular disease, 14 cases of abdominal surgery, 8 cases of severe trauma and 6 cases of poisoning. The control group consisted of 38 males and 32 females, aged 22-67 years, with an average age of (49.3±4.7) years; there were 25 cases of cardiopulmonary resuscitation, 17 cases of craniocerebral trauma and cerebrovascular disease, 11 cases of abdominal surgery, 8 cases of severe trauma and 9 cases of poisoning. There was no significant difference in age, sex and disease condition between the two groups (P>0.05); hence, the results were comparable. This study was approved by the ethics committee of our hospital, and the family members of the patients signed the informed consent.

### Preoperative Nursin:

(1) First of all, all kinds of emergency equipment and articles such as simple breathing bag, ventilator, laryngoscope, trachea cannula, sputum aspiration, sputum aspirator and vital sign monitor, a percutaneous dilational tracheostomy package, an open tracheostomy package and sedative and hemostatic drugs were prepared. (2) Psychological nursing was done for the patients who were awake before operation to gain their cooperation during the perioperative period, and midazolam or propofol was given to the patients who were nervous or agitated before operation.

### Surgical Methods:

Patients in both groups were given 10 min of conventional oxygen inhalation. They took a supine position, with the head raised and over-extended. A cotton pillow was placed under the shoulder to fully expose the neck and mark the position. Patients in the control group were given the traditional open tracheostomy. The skin, subcutaneous tissue and superficial fascia were incised longitudinally one by one in the local anesthesia. Sternohyoideus and sternothyroid were separated along the white line. The 2^nd^ and 3^rd^ tracheal ring or the 3^rd^ and 4^th^ tracheal ring was incised, and trachea cannula was inserted and fixed. Patients in the observation group underwent percutaneous dilational tracheostomy. A horizontal incision with a length of 1.0~1.5 cm was made along the skin locating point. Then a syringe containing 2 mL of 2% lidocaine was punctured in the direction which was perpendicular to the longitudinal axis of the body but slightly inclined to the foot. Negative pressure was maintained during puncture. Local anesthetic was injected for topical anesthesia if there were bubbles when the syringe was pumped back. Trocar cannula was inserted, the needle core was removed, and the guide wire was inserted for more than 10 cm towards the direction of foot. The trocar was removed after the guide wire was fixed. Pretracheal tissue and anterior tracheal wall were expanded using special dilater and dilating forceps. A trachea cannula was inserted to the trachea along the guide wire. Then the guide wire and core of the cannula were removed. The cannula was fixed, and the cuff was inflated. The trachea cannula was removed after the ventilator was connected. The direction was adjusted if there was difficulty in puncture or catheterization.

### Postoperative Nursing

High-volume, low-pressure balloon catheter with an inner diameter of 6 ~ 8 cm was used as the tracheostomy tube; the small diameter might cause sputum scab obstruction and forward incarceration of balloon, which could induce asphyxia. Therefore the change of oxygen saturation was closely observed after operation. The patients were given 0.45% saline humidification solution at a pumping speed of 3 mL/h, and they were frequently turned over. The use of high-volume, low-pressure balloon catheter avoided deflation every day. To prevent the influence of sputum scab adhesion on the tube wall on ventilation, the catheter was changed every two week. The incision with bleeding after surgery was pressed. For the incision with errhysis, suture was performed, or 1: 1000 epinephrines were dropped at the aditus antrum.

### Observational Indicators

The operation time, incision length, intraoperative bleeding volume and incision healing time of the two groups were recorded. The changes of the heart rate and oxygen saturation of the two groups during the operation were recorded. The complications of the two groups were also recorded, including bleeding (requiring blood transfusion), symptomatic tracheal stenosis, severe incision infection and severe subcutaneous emphysema.

### Statistical Analysis

Data were analyzed by SPSS 21.0. All measurement data were expressed by Mean±SD. Independent sample t test was used to compare mean values between the two groups. Paired t test was used to compare mean values in one group before and after treatment. Counting data were expressed by rate (%) and X^2^ test was used. Difference was evaluated as significant if P<0.05.

## RESULTS

### Comparison of surgical conditions between the two groups

The operation time, healing time and incision length of the observation group were shorter than those of the control group, and the intraoperative bleeding volume of the observation group was less than that of the control group; the differences were statistically significant (P<0.05, Table-I).

**Table-I:** Surgical condition between the two groups.

Group	Observation group (n=70)	Control group (n=70)	t	P
Operation time (min)	9.92±4.13	24.09±6.82	7.823	<0.05
Incision length (cm)	1.31±0.21	3.40±0.65	5.187	<0.05
Healing time (d)	6.91±0.72	7.48±0.61	3.946	<0.05
Intraoperative bleeding volume (mL)	7.81±1.83	16.34±2.83	7.605	<0.05

### Comparison of heart rate and oxygen saturation before and during operation between the two groups

There was no significant difference in the heart rate and oxygen saturation before and during operation in the observation group (P>0.05). The heart rate in the control group during operation was significantly higher than that before operation (P<0.05). There was no significant difference in oxygen saturation in the control group before and during operation (P>0.05, Table-II).

**Table-II:** Heart rate and oxygen saturation before and during operation between the two groups.

Group		Observation group (n=70)	Control group (n=70)
Heart rate (Times/min)	Before operation	135.49±15.41	134.32±18.58
	During operation	148.57±19.68	162.61±23.39*
Oxygen saturation	Before operation	60.72±12.48	60.39±11.81
	During operation	65.42±10.19	64.41±12.12

***Note:***

* means P<0.05 comparing to before operation.

### Comparison of postoperative complications between the two groups

The incidence of postoperative complications in the observation group was 24.3%, which was significantly lower than that in the control group (55.7%, P<0.05, Table-III).

**Table-III:** Postoperative complications between the two groups [n (%)].

Group	Observation group (n=70)	Control group (n=70)	X^2^	P
Bleeding (requiring blood transfusion)	0(0.0)	8(11.4)		
Symptomatic tracheal stenosis	11(15.7)	3(4.3)		
Severe incision infection	3(4.3)	14(20.0)		
Severe subcutaneous emphysema	3(4.3)	14(20.0)		
Incidence	17(24.3)	39(55.7)	8.279	<0.05

## DISCUSSION

Percutaneous dilational tracheostomy uses small incision parallel to dermatoglyph, while open tracheostomy mostly uses large longitudinal incisions to expose the field of operation.^12^-^14^ In the percutaneous dilational tracheostomy group, the trachea cannula was inserted after dilation, avoiding cutting of skin, subcutaneous tissues and tracheal rings, insertion of trachea cannula and suture; therefore the operation time was short, the trauma was small, and bleeding volume and incision infection was reduced; hence it has significant advantages in surgical incision and healing time compared to open tracheostomy,^15^ which was fully confirmed by the results of this study. In addition, the traditional tracheostomy requires specific training. The incision is long, the anterior cervical tissues need to be separated, the anterior wall of the trachea needs to be incised, and the anterior cervical muscle group needs traction during operation, which can affect the blood pressure, heart rate and oxygen saturation of patients and cause significant fluctuation of vital signs of critically ill patients.^16^ In this study, the heart rate of patients in the control group increased significantly during operation, and there was no significant difference in the oxygen saturation between the two groups before and during operation, which may be related to intraoperative oxygen inhalation.

The results of this study also showed that the incidence of complications such as bleeding, severe incision infection and severe subcutaneous emphysema in the observation group was significantly lower than that in the control group (P<0.05), which was similar to the results of Vigliaroli et al..^17^ In open tracheostomy, operation injures many tissues, some of the ligated or coagulated blood vessels falls off or opens again, and there may be chronic bleeding sometimes. In percutaneous dilational tracheostomy, tracheal cannula is wrapped because of the elastic recoil of the skin and anterior cervical tissue muscles, which can stop bleeding. In open tracheostomy, it is often necessary to ligate or cut off the isthmus of thyroid to expose the anterior wall of the trachea; however, in percutaneous dilational tracheostomy, the first or second tracheal annulus space is usually expand, mostly avoiding the isthmus of thyroid,^18^ which leads to low risk of injury in the isthmus of thyroid.

A foreign study showed that the degree of familiarity with the anatomy of cervical vessels is directly related to hemorrhage.^19^ Rajajee et al. considered that real-time ultrasound could reduce thyroid and vascular damages.^20^ In the traditional pneumotomy, the trachea and pretracheal space are large because of the extensive stripping of pretracheal fascia; gas can easily enter the anterior tracheal space and cause subcutaneous emphysema when positive pressure ventilation is used. However, the incidence of tracheal stenosis in the observation group was higher than that in the control group. It may be that the dilation and catheterization of the trachea during puncture led to the inversion of tracheal mucosa and cartilage ring into the trachea.

## CONCLUSION

In conclusion, percutaneous dilational tracheostomy is a simple and practical open airway technique because it has smaller incision, less bleeding and simpler operation than open tracheostomy. It cannot only avoid the fluctuation of vital signs of patients, but also reduce the complications of patients after operation. Generally, ICU doctors can do percutaneous dilational tracheostomy independently after simple training. But at present, percutaneous dilational tracheostomy cannot completely replace open tracheostomy. While percutaneous dilational tracheostomy is carried out, it should also have the conditions and ability to implement open tracheostomy. If percutaneous dilational tracheostomy fails, it can quickly change to open tracheostomy. In clinical work, appropriate tracheostomy should be selected according to the specific conditions of patients to minimize possible complications.

### Authors’ Contribution:

**HMG:** Study design, data collection and analysis, is responsible for integrity of research.

**HMG & HYX:** Manuscript preparation, drafting and revising.

**HMG & AHD:** Review and final approval of manuscript.
